# Discovery and Validation of an Epithelial-Mesenchymal Transition-Based Signature in Gastric Cancer by Genomics and Prognosis Analysis

**DOI:** 10.1155/2021/9026918

**Published:** 2021-10-26

**Authors:** Huiyong Xu, Huilai Wan, Maoshu Zhu, Lianghua Feng, Hui Zhang, Fengbing Su

**Affiliations:** The Fifth Hospital of Xiamen, Xiamen, 361101 Fujian, China

## Abstract

**Objective:**

Epithelial-mesenchymal transition (EMT) exerts a key function in cancer initiation and progression. Herein, we aimed to develop an EMT-based prognostic signature in gastric cancer.

**Methods:**

The gene expression profiles of gastric cancer were obtained from TCGA dataset as a training set and GSE66229 and GSE84437 datasets as validation sets. By LASSO regression and Cox regression analyses, key prognostic EMT-related genes were screened for developing a risk score (RS) model. Potential small molecular compounds were predicted by the CMap database based on the RS model. GSEA was employed to explore signaling pathways associated with the RS. ESTIMATE and seven algorithms (TIMER, CIBERSORT, CIBERSORT-ABS, QUANTISEQ, MCPCOUNTER, XCELL, and EPIC) were applied to assess the RS and immune microenvironment.

**Results:**

This study developed an EMT-related gene signature comprised of SERPINE1, PCOLCE2, MATN3, and DKK1. High-RS patients displayed poorer survival outcomes than those with low RS. ROC curves demonstrated the robustness of the model in predicting the prognosis. After external validation, the RS model was an independent risk factor for gastric cancer. Several compounds were predicted for gastric cancer treatment based on the RS model. ECM receptor interaction, focal adhesion, pathway in cancer, TGF-beta, and WNT pathways were distinctly activated in high-RS samples. Also, high RS was significantly associated with increased stromal and immune scores and increased infiltration of CD4+ T cell, CD8+ T cell, cancer-associated fibroblast, and macrophage in gastric cancer tissues.

**Conclusion:**

Our findings suggested that the EMT-related gene model may robustly predict gastric cancer prognosis, which could improve the efficacy of personalized therapy.

## 1. Introduction

Gastric cancer represents a common aggressive malignancy and a common cause of cancer-related deaths globally due to its rapid progress to advanced stages and badly metastatic characteristics [1]. The incidence and prevalence of gastric cancer vary geographically [2]. Despite the improvement in clinical outcomes by implementing standard D2 lymphadenectomy as well as development of chemotherapy and targeted therapy, the overall survival rate of gastric cancer patients is <30% [3]. As a heterogeneous malignancy [4], survival outcomes may greatly vary even for subjects with similar clinical characteristics and therapy regimens, indicating that traditional clinicopathologic characteristics are inadequate for prognosis prediction and risk stratification [5]. Hence, it is important to develop novel clinical tools for predicting the prognosis of gastric cancer.

Epithelial-mesenchymal transition (EMT), a well-characterized embryological process, is a critical molecular step during the process of distant metastases [6–8]. Clinically, EMT is in relation to unfavorable survival outcomes of gastric cancer [9]. During the EMT process, gastric cancer cells lose the expression of cellular adhesion proteins like E-cadherin and tight junction proteins as well as express many mesenchymal markers like N-cadherin, Vimentin, and ZEB1 [10]. The mesenchymal phenotype also may raise resistance to chemotherapy and contribute to a desirable prognosis [11]. Therefore, an in-depth comprehension on the mechanisms of the EMT process in gastric cancer is required for promoting the progress of specific treatment strategies. Because various large datasets are easily accessible, exploring the gene signatures underlying the mechanisms of gastric cancer has flourished [12–14]. Despite the extensive research on the mechanisms of EMT in gastric cancer, the prognostic value of EMT-related genes is still inconclusive. Hence, this study constructed an EMT-based signature for predicting survival outcomes of gastric cancer patients. After external verification, this signature might be a robust prognostic prediction tool and assist clinical strategy.

## 2. Materials and Methods

### 2.1. Gene Expression Profiles and Data Processing

RNA-sequencing (RNA-seq) profiles of 32 normal samples and 350 gastric cancer samples were downloaded from The Cancer Genome Atlas (TCGA) via Genomic Data Commons (GDC; https://portal.gdc.cancer.gov/). Also, the matched clinical information was also retrieved. RNA-seq data were converted to transcripts per kilobase million (TPM) values. This dataset was used as the training set. From the Gene Expression Omnibus (GEO; http://www.ncbi.nlm.nih.gov/geo/), microarray expression profiling and clinical information of 400 cases of gastric cancer were retrieved from the GSE66229 dataset on the GPL570 platform ([HG-U133_Plus_2] Affymetrix Human Genome U133 Plus 2.0 Array) [15]. Furthermore, expression profiles and clinical features of 433 gastric cancer were obtained from the GSE84437 dataset on the GPL6947 platform (Illumina HumanHT-12 V3.0 expression beadchip) [16]. The raw microarray data were adjusted by background, normalized, and log transformed. The GSE66229 and GSE84437 datasets were employed as the validation sets. The “HALLMARK_EPITHELIAL_MESENCHYMAL_TRANSITION” gene set was retrieved from the Gene Set Enrichment Analysis (GSEA) database (http://software.broadinstitute.org/gsea/index.jsp) [17] (Supplementary Table 1).

### 2.2. Differential Expression Analysis

The expression of EMT-related genes in 350 gastric cancer tissue specimens was compared with 32 normal tissues in TCGA dataset using the limma package [18]. The ∣log fold‐change | >1 and adjusted *p* < 0.05 were set as cutoff criteria. Differentially expressed EMT-related genes were visualized into volcano plots and heatmaps.

### 2.3. Functional and Pathway Enrichment Analysis

Biological functions of differentially expressed EMT-related genes were analyzed via the clusterProfiler package, containing Gene Ontology (GO) and Kyoto Encyclopedia of Genes and Genomes (KEGG) pathway enrichment analysis [19]. Terms with false discovery rate (FDR) < 0.05 were significantly enriched.

### 2.4. Small Molecular Compound Prediction

Differentially expressed genes with ∣log fold‐change | >1 and adjusted *p* < 0.05 were screened between the high- and low-RS groups. Then, up- and downregulated tags were separately uploaded onto Connectivity Map (CMap) [20]. The match between these genes and small molecular compounds from CMap was evaluated through a connectivity score from −1 to 1. Positive scores denote stimulative effects of compounds on the query signatures. Meanwhile, negative scores implicate inhibitory effects of compounds on the query signatures.

### 2.5. Generation and Verification of a Risk Score (RS) Model

In TCGA dataset, differentially expressed EMT-related genes with prognostic value were filtered via univariate Cox regression analyses. Genes with *p* < 0.05 were included for least absolute shrinkage and selection operator (LASSO) Cox regression model analyses using the glmnet package [21]. The penalized Cox regression model with LASSO penalty was employed for achieving shrinkage and variable selection. Tenfold cross-validation was presented for determining the optimal value of the penalty parameter *λ*. Based on *λ* value, factors with the matched coefficients were chosen. RS of each patient was determined on the basis of the expression levels of genes and their coefficients. According to the median value, patients were split into the high- and low-RS groups. Kaplan-Meier curves and log-rank test were employed for analyzing the overall survival (OS) difference between the high- and low-RS groups. Receiver operating characteristic (ROC) analysis was conducted for detecting the predictive accuracy of this RS model in the prognosis. Furthermore, the RS model was externally validated in the GSE66229 and GSE84437 datasets.

### 2.6. Screening Independent Prognostic Factors

Univariate Cox regression analysis was applied for evaluating the significance of the RS model and clinical characteristics in predicting gastric cancer patients' OS. Factors with *p* < 0.05 were included for multivariable logistic regression analysis, and confounding factors were excluded. The hazard ratio (HR) and 95% confidence interval (CI) were calculated. The results were visualized into a forest plot.

### 2.7. Subgroup Analysis

To evaluate the predictive sensitivity of the RS model in gastric cancer OS, patients were split into subgroups based on clinical features, as follows: age (>65 and ≤65), gender (female and male), M (M0 and M1), N (N0 and N1-3), T (T1-2 and T3-4), and stage (I-II and III-IV). The survival difference between the high- and low-RS samples was compared in each subgroup.

### 2.8. Development of a Prognostic Nomogram

RS and traditional clinicopathological characteristics were included in the nomogram through the rms package. To assess the performance of the nomogram in predicting 1-, 3-, and 5-year OS time, nomogram-predicted OS probability was compared with actual survival time by calibration curves. Furthermore, the predictive efficacy of this nomogram was externally verified in the GSE66229 and GSE84437 datasets.

### 2.9. GSEA

The GSEA method was applied for exploring the potential KEGG pathways activated in high-RS gastric cancer samples. The reference gene set was retrieved from “c2.cp.kegg.v7.1.symbols” file. The significantly enriched pathways were screened with FDR < 0.05.

### 2.10. Estimation of Immune Score, Stromal Score, and Tumor Purity

The immune score, stromal score, and tumor purity were estimated in gastric cancer tissue specimens via the Estimation of STromal and Immune cells in MAlignant Tumor tissues using Expression data (ESTIMATE) algorithm [22].

### 2.11. Analysis of Immune Cell Infiltrations

To reveal the associations of the risk score and diverse tumor-infiltrating immune cells, seven algorithms including TIMER, CIBERSORT, CIBERSORT-ABS, QUANTISEQ, MCPCOUNTER, XCELL, and EPIC were applied for quantifying the infiltration levels. Differences in immune-infiltrating cell fractions were estimated between the high- and low-risk groups.

### 2.12. Statistical Analysis

All statistical analyses were conducted using R software (version 3.6.2; https://www.r-project.org/). Comparisons between groups were carried out with Student's *t*-test and Wilcoxon rank-sum test. The Spearman correlation test was applied to assess the correlation between immune cells. *p* values < 0.05 were considered statistically significant.

## 3. Results

### 3.1. Identification of Dysregulated EMT-Related Genes and Their Functions in Gastric Cancer

**Figure 1:**
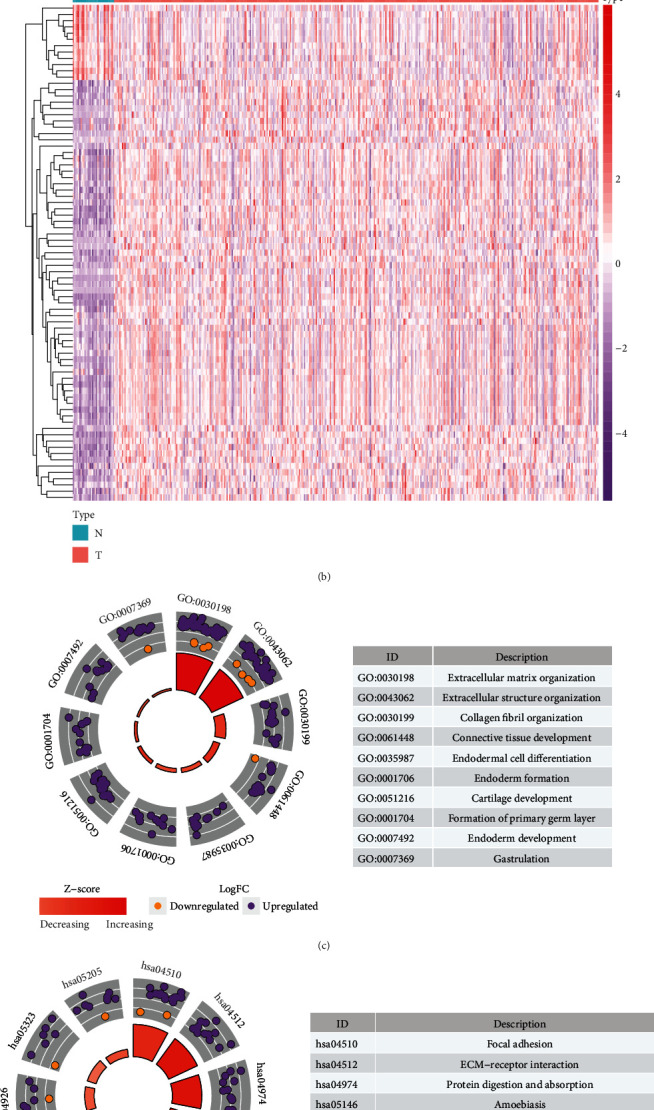
Identification of dysregulated EMT-related genes, biological functions in gastric cancer. (a) Volcano plot depicting the dysregulated EMT-related genes between gastric cancer and normal tissues. *X*-axis represents log fold-change, and *Y*-axis indicates -log10 (adjusted *p* value). Red and green dots represent up- and downregulated EMT-related genes in gastric cancer, and black dots represent no significant genes. (b) Heatmaps for dysregulated EMT-related genes between tumor and normal tissues. *X*-axis represents the sample type, and *Y*-axis depicts dysregulated EMT-related genes. Red and blue show up- and downregulation in gastric cancer, respectively. (c, d) The top ten GO and KEGG terms enriched by dysregulated EMT-related genes.

Following the comparison of expression of EMT-related genes between gastric cancer and normal tissues, 79 differentially expressed EMT-related genes with ∣log fold‐change | >1 and adjusted *p* < 0.05 were identified (Supplementary Table 2). Among them, 67 EMT-related genes were upregulated and 12 were downregulated in gastric cancer (Figures 1(a) and 1(b)). GO enrichment analyses were conducted to elucidate the functional characteristics of these differentially expressed EMT-related genes. Our data showed that these genes were markedly enriched in extracellular matrix (ECM) organization, extracellular structure organization, and collagen fibril organization (Figure 1(c)). Meanwhile, these genes were distinctly related to several key pathways like focal adhesion, ECM-receptor interaction, PI3K-Akt signaling pathway, and proteoglycans in cancer (Figure 1(d)). Hence, it is required to illustrate their clinical implications in gastric cancer.

### 3.2. Generation of a Prognostic EMT-Related RS Model for Gastric Cancer

**Figure 2:**
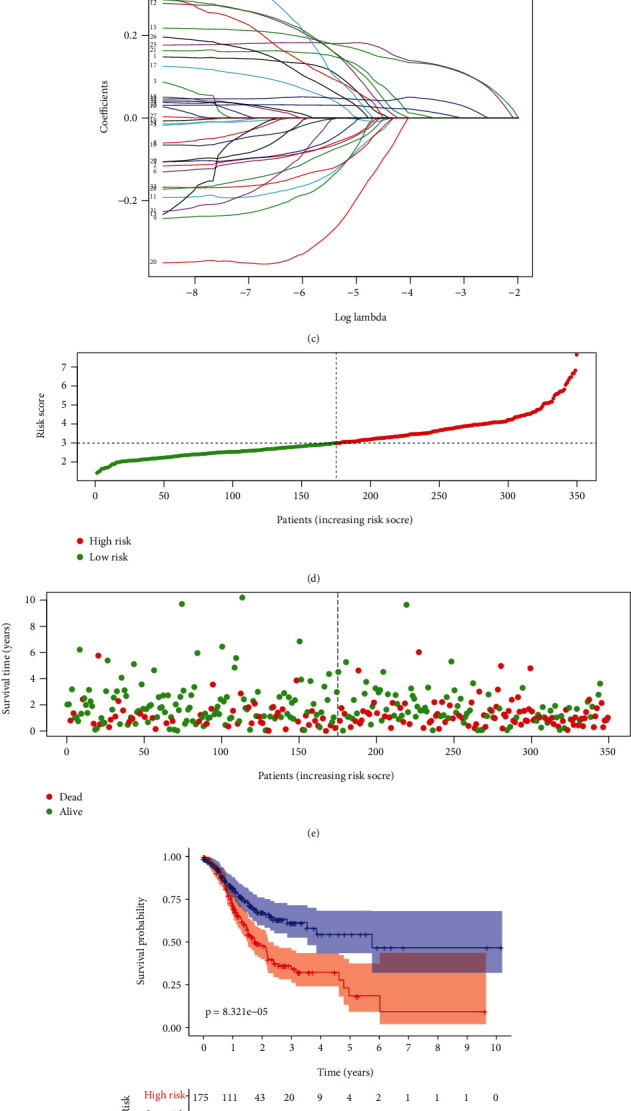
Generation of a prognostic EMT-related gene model for gastric cancer in TCGA dataset. (a) Univariate Cox regression analysis for prognosis-related EMT-related genes in gastric cancer. (b) Selecting the optimal parameter (*λ*) in the LASSO model using 10-fold cross-verification. (c) LASSO coefficient profiles of prognosis-related EMT genes. (d) Distribution of RS in gastric cancer patients and determination of the cutoff value of high-RS (red) and low-RS (green) groups according to RS median. (e) Distribution of survival status (dead: red and alive: green) in high- and low-RS groups. (f) Kaplan-Meier OS curves for the high- and low-RS groups. (g) The time-dependent ROC for the RS model. (h) Univariate and (i) multivariate Cox regression analyses of RS and other clinical features.

By the mRNA expression profiling of TCGA dataset, we screened 35 EMT-related genes associated with OS of gastric cancer with univariable Cox regression analysis (Figure 2(a); Table 1). These genes were further analyzed using LASSO Cox regression model analysis. As a result, we generated a 4-EMT-related gene model for gastric cancer (Figures 2(b) and 2(c)). The RS was determined for each gastric cancer, as follows: RS = 0.127258355254692∗SERPINE1 expression + 0.04303645817321∗PCOLCE2 expression + 0.128510051263955∗MATN3 expression + 0.0116209970037921∗DKK1 expression. Because the median RS was convenient for clinical application, this study set the median value as the cutoff value, and patients were split into the high- and low-RS groups (Figure 2(d)). We compared the survival status between groups. In Figure 2(e), more deaths occurred in the high-RS group. Furthermore, for each patient, high RS was indicative of an unfavorable prognosis (*p* = 8.321*e* − 05; Figure 2(f)). However, there was no significant difference in clinical characteristics between the high- and low-RS groups (Table 2). The area under the curve (AUC) of the RS model was 0.763, indicating good performance in predicting patients' OS (Figure 2(g)). Our univariate Cox regression analysis showed that age (*p* = 0.033), stage (*p* = 0.002), N (*p* = 0.022), and RS (*p* < 0.001) were distinctly associated with a poor prognosis (Figure 2(h)). Under multivariate Cox regression analysis, age (*p* = 0.004), stage (*p* = 0.005), and RS (*p* < 0.001) were independent risk factors for the gastric cancer prognosis (Figure 2(i)).

### 3.3. Subgroup Analysis of the Prognostic Value of the EMT-Related RS Model

**Figure 3:**
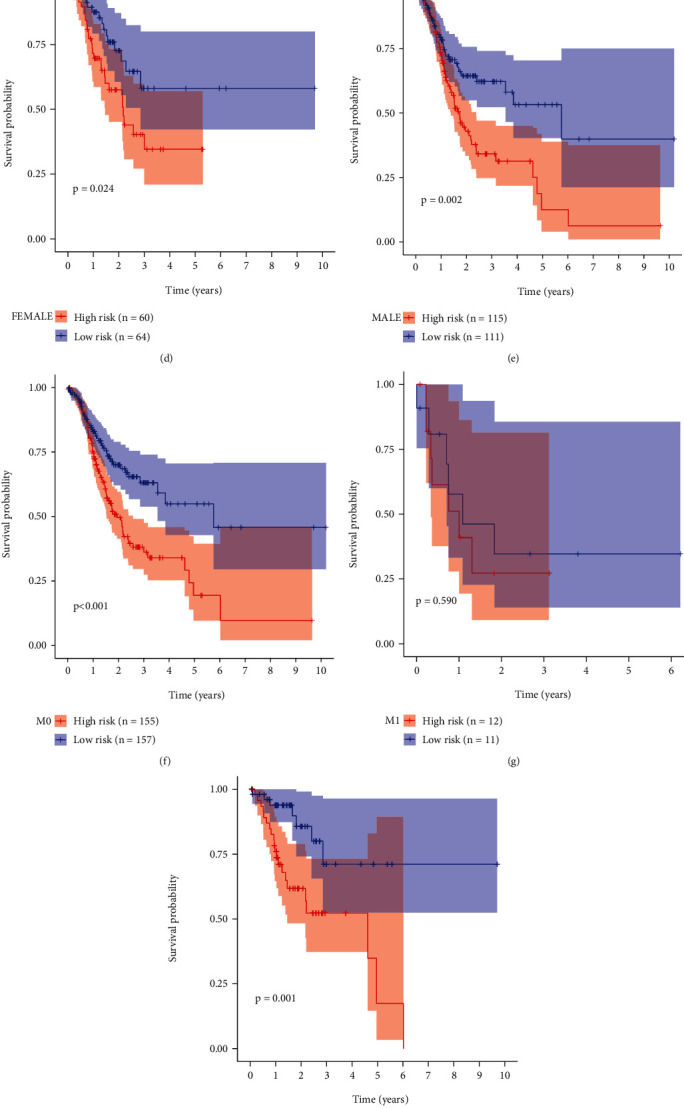
Subgroup analysis of the prognostic value of the EMT-related RS model. (a) Heatmap of the expression of SERPINE1, PCOLCE2, MATN3, and DKK1 in high- and low-RS groups. Red and green show up- and downregulation. Kaplan-Meier curves between high- and low-RS gastric cancer patients in different subgroups including (b) age ≥ 65 and (c) age < 65; (d) female and (e) male; (f) M0 and (g) M1; (h) N0 and (i) N1-3; (j) T1-2 and (k) T3-4; (l) stage I-II and (m) stage III-IV.

SERPINE1, PCOLCE2, MATN3, and DKK1 expression was compared between the high- and low-RS groups. In Figure 3(a), there were increased expression levels in the high- than low-RS groups. To assess whether the EMT-related RS model could sensitively predict gastric cancer patients' prognosis, we carried out subgroup analysis. Our data showed that high RS was predictive of undesirable survival outcomes compared with low RS in each subgroup including age ≥ 65 (*p* = 0.002; Figure 3(b)) and age < 65 (*p* = 0.009; Figure 3(c)), female (*p* = 0.024; Figure 3(d)) and male (*p* = 0.002; Figure 3(e)), M0 (*p* < 0.001; Figure 3(f)) and M1 (*p* = 0.590; Figure 3(g)), N0 (*p* = 0.001; Figure 3(h)) and N1-3 (*p* = 0.005; Figure 3(i)), T1-2 (*p* = 0.003Figure 3(j)) and T3-4 (*p* = 0.006; Figure 3(k)), stage I-II (*p* < 0.001; Figure 3(l)) and stage III-IV (*p* = 0.042; Figure 3(m)).

### 3.4. External Validation of the EMT-Related RS Model

**Figure 4:**
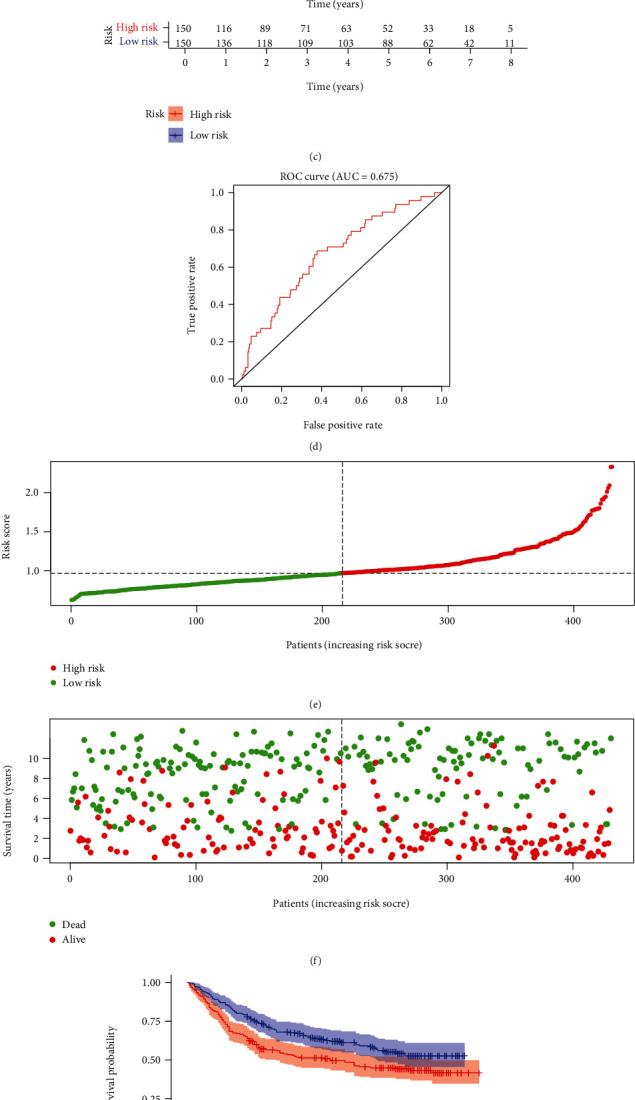
External validation of the EMT-related RS model in GSE66229 and GSE84437 datasets. (a) Distribution of RS in gastric cancer samples and determination of the cutoff value of high-RS (red) and low-RS (green) groups according to RS median in the GSE66229 dataset. (b) Distribution of survival status (red: dead and green: alive) in high- and low-RS groups in GSE66229 dataset. (c) Kaplan-Meier OS curves of high- and low-RS groups in GSE66229 dataset. (d) ROC curves of the RS model in GSE66229 dataset. (e) Distribution of RS in gastric cancer samples and determination of the cutoff value of high-RS (red) and low-RS (green) groups according to RS median in GSE84437 dataset. (f) Distribution of survival status (red: dead and green: alive) in high- and low-RS groups in GSE84437 dataset. (g) Kaplan-Meier OS curves of high- and low-RS groups in GSE84437 dataset. (h) ROC curves of the RS model in GSE84437 dataset.

**Figure 5:**
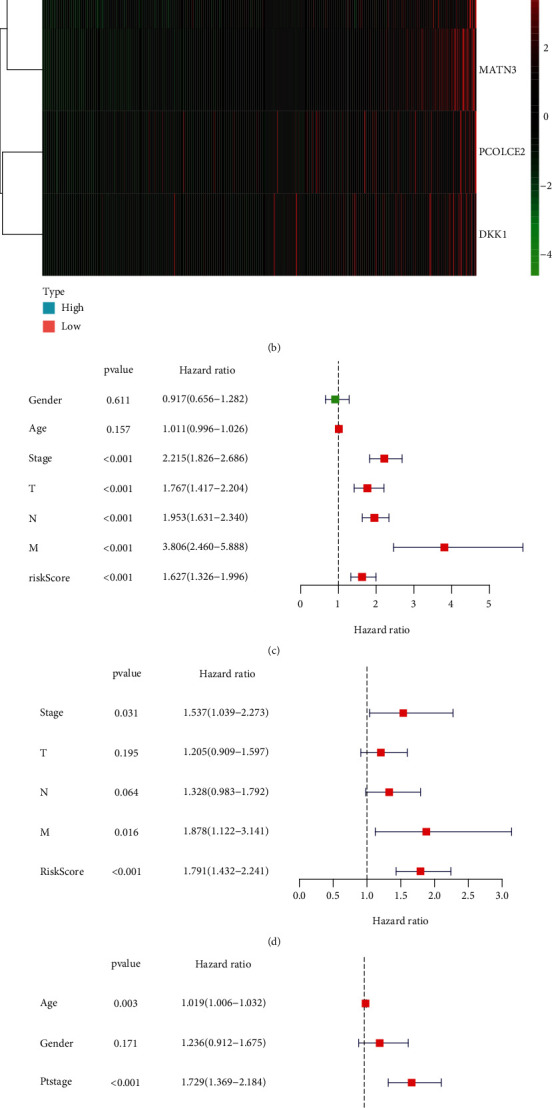
External validation of the independency of the EMT-related RS model in predicting prognosis in GSE66229 and GSE84437 datasets. (a, b) Heatmap of the expression of SERPINE1, PCOLCE2, MATN3, and DKK1 in high- and low-RS groups in (a) GSE66229 and (b) GSE84437 datasets. Red and green indicate up- and downregulation. (c) Univariate and (d) multivariate Cox regression analyses of the RS model and other clinicopathological characteristics in GSE66229 dataset. (e) Univariate and (f) multivariate Cox regression analyses of the RS model and other clinicopathological characteristics in GSE84437 dataset.

The predictive efficacy of the EMT-related RS model was externally verified in the GSE66229 and GSE84437 datasets. With the same formula, we calculated the RS of each patient. In the GSE66229 dataset, patients were split into the high- and low-RS groups based on the median value (Figure 4(a)). As expected, more deaths were found in the high-RS group (Figure 4(b)). The clinical features between groups were compared, and we found that high RS was in relation to late stage, T, and M (Table 3). Furthermore, high-RS patients exhibited more undesirable survival outcomes (*p* = 7.802*e* − 07; Figure 4(c)). AUC of the RS model was 0.675 (Figure 4(d)). Similarly, we split patients in the GSE84437 dataset into the high- and low-RS groups (Figure 4(e)). There were more patients with dead status in the high-RS group (Figure 4(f)). In Figure 4(g), high RS was distinctly related to poor prognosis (*p* = 5.333*e* − 03). And AUC of the model was 0.637 (Figure 4(h)). Consistent with TCGA dataset, increased SERPINE1, PCOLCE2, MATN3, and DKK1 expression was detected in the high-RS group than the low-RS group in GSE66229 (Figure 5(a)) and GSE84437 (Figure 5(b)) datasets. Following univariate (Figure 5(c)) and multivariate (Figure 5(d)) Cox regression analyses, the RS model was markedly correlated with gastric cancer prognosis in the GSE66229 dataset. Consistently, in the GSE84437 dataset, the RS model was also a risk factor for prognosis according to univariate (Figure 5(e)) and multivariate (Figure 5(f)) Cox regression analyses. Collectively, the EMT-related RS model displayed good generalizability in clinical practice.

### 3.5. Development of a Prognostic Nomogram Based on the EMT-Related RS Model

**Figure 6:**
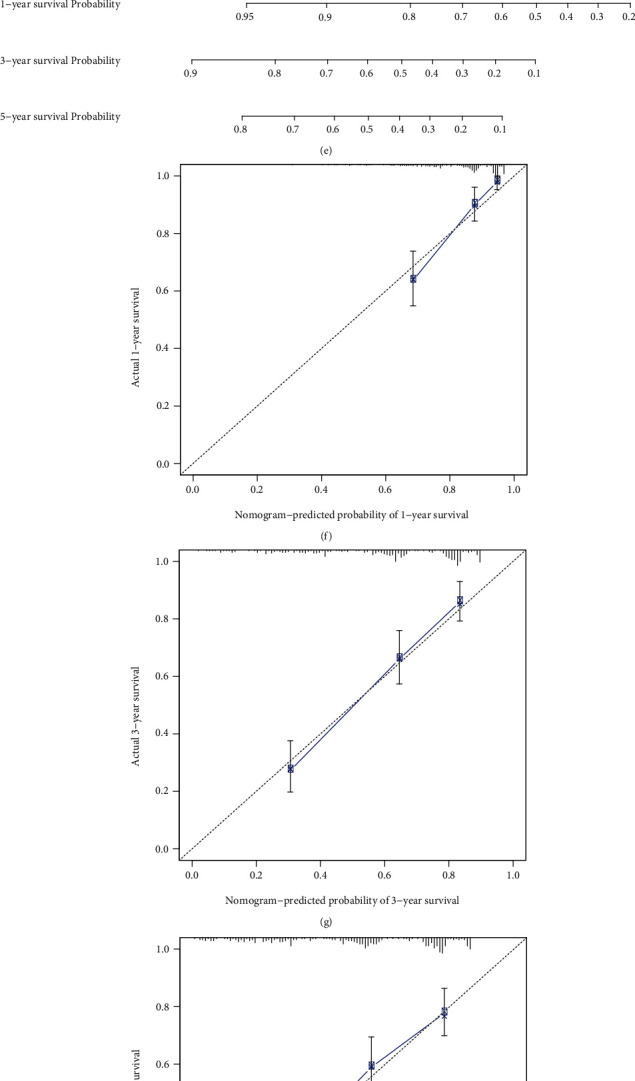
Discovery and verification of a prognostic nomogram based on the EMT-related RS model. (a) Establishment of a prognostic nomogram in TCGA dataset. (b–d) The calibration curves for the relationships between the nomogram-predicted and actual 1-, 3-, and 5-year survival probabilities. (e) Validation of the prognostic nomogram in GSE66229 dataset and (f–h) the calibration curves for the relationships between the nomogram-predicted and actual 1-, 3-, and 5-year survival probabilities. (i) Validation of the prognostic nomogram in GSE84437 dataset and (j–l) the calibration curves for the relationships between the nomogram-predicted and actual 1-, 3-, and 5-year survival probabilities.

Independent risk factors were included in the prognostic nomogram for gastric cancer. In TCGA dataset, the nomogram including age, stage, and RS was constructed for predicting patients' survival duration (Figure 6(a)). The calibration curves confirmed that the nomogram-predicted 1-, 3-, and 5-year survival probabilities were in accord with observed survival duration (Figures 6(b)–6(d)). Similarly, the nomogram was developed in the GSE66229 dataset (Figure 6(e)). The well predictive efficacy was verified by the calibration curves (Figures 6(f)–6(h)). Meanwhile, the nomogram was validated in the GSE84437 dataset (Figures 6(i)–6(l)).

### 3.6. Prediction of Underlying Small Molecular Compounds for Gastric Cancer Based on Dysregulated EMT-Related Genes

**Figure 7:**
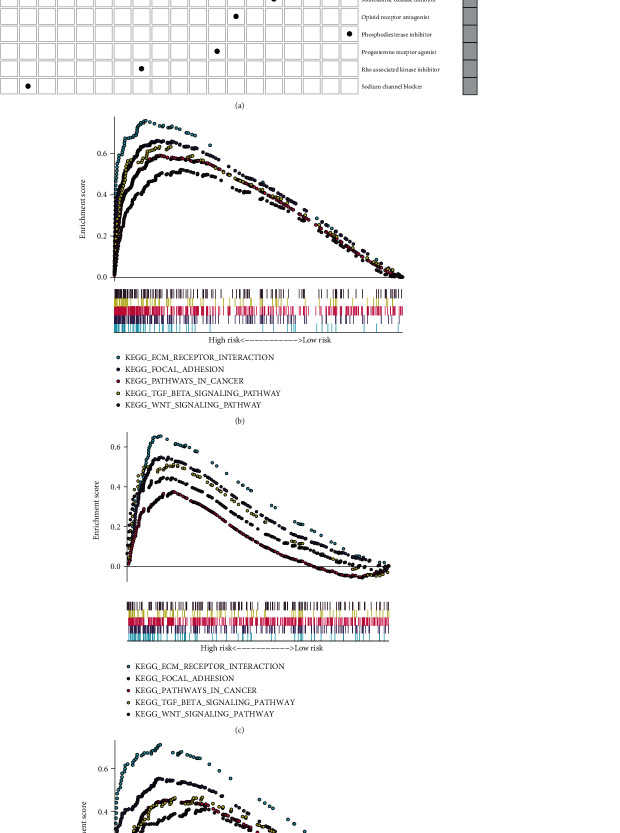
Screening potential small molecular compounds and activated pathways associated with RS model in gastric cancer. (a) Candidate small molecular compounds that were predicted by the CMAP database based on differentially expressed EMT-related genes. *X*-axis shows mechanism of action, and *y*-axis represents small compounds. (b–d) Activated pathways in high-RS gastric cancer samples in (b) TCGA, (c) GSE66229, and (d) GSE84437 datasets.

Totally, 209 differentially expressed genes were identified between the high- and low-RS groups (Supplementary Table 3). Based on them, underlying compounds were predicted by the CMap database, as listed in Table 4. The mechanism of action analysis was then conducted to investigate the shared mechanisms among the compounds. In Figure 7(a), estrogen receptor agonist was shared by dienestrol and diethylstilbestrol.

### 3.7. Identification of the EMT-Related Gene Model Associated Signaling Pathways

In TCGA dataset, ECM receptor interaction (NES = 2.24, FDR = 0.004), focal adhesion (NES = 2.13, FDR = 0.007), pathway in cancer (NES = 2.06, FDR = 0.011), TGF-beta signaling pathway (NES = 2.01, FDR = 0.011), and Wnt signaling pathway (NES = 1.79, FDR = 0.033) were markedly activated in high-RS gastric cancer specimens (Figure 7(b)). The above activated pathways were confirmed in the GSE66229 (Figure 7(c)) and GSE84437 (Figure 7(d)) datasets.

### 3.8. Associations between the EMT-Related RS Model and Immune Microenvironment of Gastric Cancer

**Figure 8:**
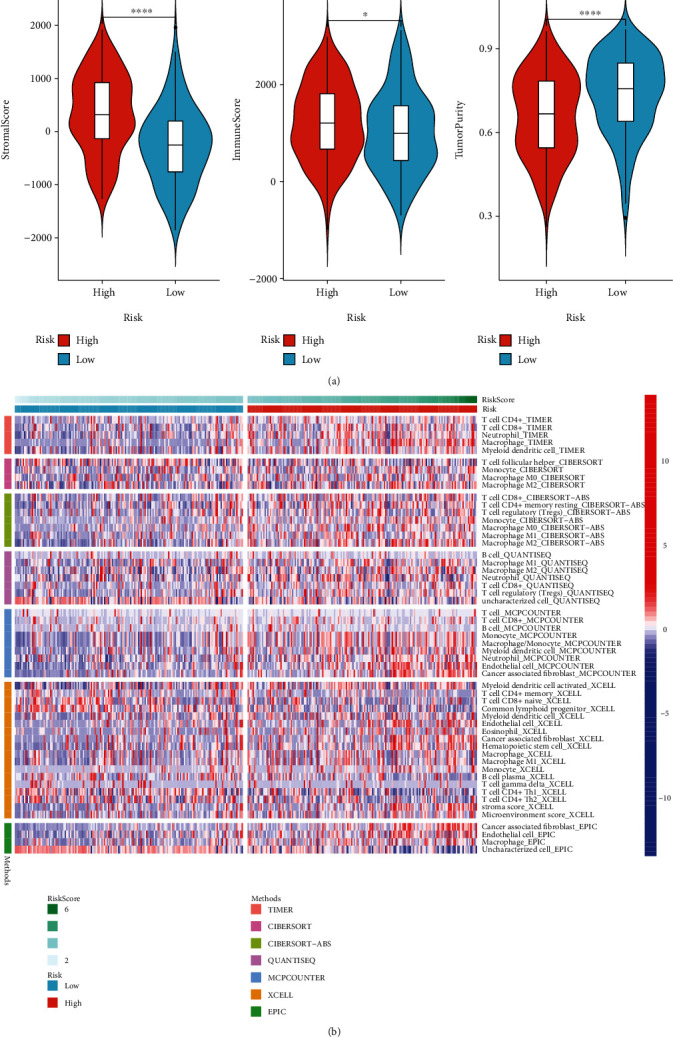
The relationships between the EMT-related RS model and immune microenvironment of gastric cancer. (a) Violin plots of stromal score, immune score, and tumor purity in high- and low-RS groups. (b) Heatmap showing infiltration levels of immune cells in high- and low-RS groups using seven algorithms including TIMER, CIBERSORT, CIBERSORT-ABS, QUANTISEQ, MCPCOUNTER, XCELL, and EPIC. ^∗^*p* < 0.05; ^∗∗∗∗^*p* < 0.0001.

Using the ESTIMATE algorithm, we estimated the stromal score, immune score, and tumor purity of gastric cancer tissues from TCGA dataset and analyzed their relationships with the RS. Our data showed that high RS was distinctly related to increased stromal and immune scores as well as lowered tumor purity in gastric cancer (Figure 8(a)). Seven algorithms including TIMER, CIBERSORT, CIBERSORT-ABS, QUANTISEQ, MCPCOUNTER, XCELL, and EPIC were employed to estimate the immune cell infiltrations in each sample. We compared the differences in immune cell infiltrations between the high- and low-RS groups. In Figure 8(b), higher infiltration levels of CD4+ T cell, CD8+ T cell, cancer-associated fibroblast, and macrophage were found in the high-RS group than the low-RS group.

## 4. Discussion

EMT-based gene signatures have been developed in bladder cancer [23], glioma [24], and colorectal cancer [25]. EMT is determined to be closely associated with gastric cancer progression and prognosis. Increased motility and invasiveness mediated by the EMT process are key during the initiation of cancer metastasis. However, no studies have reported the prognostic value of EMT-based signatures in gastric cancer. Here, we developed an EMT-related RS model that was comprised of SERPINE1, PCOLCE2, MATN3, and DKK1 in gastric cancer via the LASSO method, which may classify gastric cancer patients into the high- and low-risk categories. This LASSO method has been widely applied for analyzing high-dimensional data, which may screen feature signatures with robust prognostic potential and weak correlations among them to avoid overfitting [26].

Alterations in gene expression are in relation to the carcinogenic process. Here, we screened 67 upregulated and 12 downregulated EMT-related genes in gastric cancer. These genes were distinctly enriched in ECM organization, extracellular structure organization, and collagen fibril organization as well as several cancer-related pathways like focal adhesion, ECM-receptor interaction, PI3K-Akt signaling pathway, and proteoglycans in cancer, highlighting their critical implications in gastric cancer pathogenesis. By the LASSO method, we generated an EMT-based signature containing SERPINE1, PCOLCE2, MATN3, and DKK1. After validation, this signature was independently predictive of survival outcomes. Previously, SERPINE1 upregulation was found in gastric cancer and in relation to unfavorable prognoses [27]. Furthermore, it was tightly correlated to the EMT process in gastric cancer [28]. As an oncogene, it may facilitate tumor cell proliferation, migration, and invasion in gastric cancer through mediating the EMT process [29]. The roles of SERPINE1 on angiogenesis and metastasis in gastric cancer were also found [30]. MATN3 was aberrantly methylated and dysregulated in gastric cancer and related to an undesirable prognosis [31]. DKK1, as an inhibitor of Wnt signaling, was also in relation to survival outcomes of gastric cancer [32]. Nevertheless, more research should be conducted for investigating the roles of PCOLCE2 in gastric cancer progression. To facilitate personalized prediction of the patient's prognosis, we generated the nomogram by incorporating the RS model and traditional clinicopathological characteristics. These model-predicted survival probabilities were highly consistent with actual survival probabilities.

Several small molecular compounds were predicted for treating gastric cancer based on the RS model such as puromycin, trolox C, cloxacillin, indoprofen, diethylstilbestrol, and caffeic acid. In our future studies, we will verify the therapeutic effects of these compounds on antigastric cancer by experiments. Our GSEA demonstrated that ECM receptor interaction, focal adhesion, pathway in cancer, TGF-beta signaling pathway, and Wnt signaling pathway were markedly activated in high-RS gastric cancer, indicating that this model was in relation to these pathways. The immune microenvironment exerts a key role in tumor progression. Our further analysis found tight associations between this model and immune microenvironment. This indicated that EMT might participate in reshaping the immune microenvironment of gastric cancer, which will be validated in our future research.

## 5. Conclusion

Collectively, our study established an EMT-based signature that may robustly predict gastric cancer prognosis and improve the efficacy of personalized therapy. The predictive performance will be verified in a larger cohort of gastric cancer.

## Figures and Tables

**Table 1:** Prognosis-related EMT-related gene signatures for gastric cancer by univariate Cox regression analysis.

ID	HR	HR.95L	HR.95H	*p* value	ID	HR	HR.95L	HR.95H	*p* value
CTHRC1	1.200409	1.06768	1.349637	0.002248	THBS2	1.119698	1.015975	1.234009	0.022637
INHBA	1.177176	1.033914	1.340289	0.013751	SFRP1	1.090971	1.002915	1.186759	0.042586
COL1A1	1.12936	1.013576	1.25837	0.027503	COL5A1	1.142209	1.006216	1.296582	0.039804
BGN	1.179647	1.039263	1.338995	0.010597	LOX	1.25252	1.090202	1.439005	0.001475
COL4A1	1.215442	1.027667	1.437527	0.022685	PCOLCE2	1.249504	1.085789	1.437903	0.001879
TIMP1	1.186359	1.00804	1.396222	0.039751	CDH11	1.208569	1.052499	1.387781	0.007247
COL5A2	1.193086	1.03962	1.369208	0.011969	SFRP4	1.078954	1.005406	1.157882	0.034891
THY1	1.204512	1.039918	1.395158	0.013062	MATN3	1.278741	1.131943	1.444577	7.75E-05
FAP	1.167508	1.031661	1.321244	0.014135	NID2	1.235369	1.057539	1.443103	0.007689
COL3A1	1.150473	1.027265	1.288458	0.015291	MYL9	1.093798	1.005017	1.190421	0.037909
CALU	1.260293	1.001195	1.586444	0.048823	FN1	1.124577	1.018254	1.242003	0.020507
ADAM12	1.183344	1.044276	1.340931	0.008311	PRRX1	1.140897	1.011825	1.286434	0.031407
COL1A2	1.151805	1.024302	1.29518	0.018221	LUM	1.19584	1.054413	1.356237	0.005352
SPARC	1.263289	1.09289	1.460256	0.00157	DCN	1.159358	1.031313	1.303301	0.013275
SERPINE1	1.24028	1.117038	1.377119	5.51E-05	FBLN1	1.110247	1.017339	1.21164	0.019002
PDGFRB	1.189439	1.028726	1.375258	0.019162	MFAP5	1.117742	1.010726	1.236089	0.030178
VCAN	1.23074	1.079319	1.403403	0.001938	ACTA2	1.119472	1.016273	1.23315	0.02219
DKK1	1.067624	1.002775	1.136667	0.040693					

**Table 2:** Clinical characteristics of high- and low-RS gastric cancer patients in TCGA dataset.

Characteristics		High risk (*N* = 175)	Low risk (*N* = 175)	Total (*N* = 350)	*p* value
Age	<65	81	69	150	0.2348
	≥65	94	106	200	
Stage	Stage I	21	28	49	0.619
	Stage II	55	56	111	
	Stage III	79	76	155	
	Stage IV	20	15	35	
T	T1	3	13	16	0.0757
	T2	39	35	74	
	T3	78	83	161	
	T4	52	43	95	
	TX	3	1	4	
M	M0	155	157	312	0.9404
	M1	12	11	23	
	MX	8	7	15	
N	N0	49	55	104	0.8117
	N1	45	48	93	
	N2	36	36	72	
	N3	40	31	71	
	NX	5	5	10	
Gender	Female	60	64	124	0.7374
	Male	115	111	226	
Grade	G1	4	5	9	0.9717
	G2	62	63	125	
	G3	104	103	207	
	GX	5	4	9	

**Table 3:** Clinical characteristics of gastric cancer patients in the GSE66229 dataset.

Characteristics		High risk (*N* = 150)	Low risk (*N* = 150)	Total (*N* = 300)	*p* value
Age	<65	87	74	161	0.1647
	≥65	63	76	139	
Stage	Stage I	9	21	30	0.0073
	Stage II	40	56	96	
	Stage III	55	40	95	
	Stage IV	45	32	77	
	NA	1	1	2	
T	T2	75	111	186	<0.0001
	T3	60	31	91	
	T4	14	7	21	
	NA	1	1	2	
M	M0	131	142	273	0.0437
	M1	19	8	27	
N	N0	14	24	38	0.1309
	N1	62	69	131	
	N2	47	33	80	
	N3	27	24	51	
Gender	Female	53	48	101	0.6251
	Male	97	102	199	

**Table 4:** Potential small compounds for treating gastric cancer based on dysregulated EMT-related genes.

Rank	CMap name	Mean	*n*	Enrichment	*p*	Specificity	Percent nonnull
1	Puromycin	0.694	4	0.929	0.00004	0.0562	100
2	Trolox C	0.461	4	0.89	0.00014	0	75
3	Cloxacillin	-0.487	4	-0.869	0.0006	0	75
4	Indoprofen	-0.307	4	-0.815	0.00213	0.0333	50
5	Diethylstilbestrol	-0.338	6	-0.663	0.00407	0.0082	50
6	Caffeic acid	0.398	3	0.853	0.00605	0	66
7	Benzamil	-0.302	6	-0.629	0.0081	0	50
8	STOCK1N-35874	-0.613	2	-0.916	0.01447	0.0331	100
9	Fasudil	-0.469	2	-0.904	0.01863	0	100
10	Amrinone	0.51	4	0.688	0.01975	0.0147	75
11	5155877	0.419	4	0.675	0.02441	0.1313	75
12	Eticlopride	-0.279	4	-0.673	0.0257	0.0758	50
13	Meropenem	0.309	4	0.668	0.02711	0.0163	50
14	16-Phenyltetranorprostaglandin E2	-0.486	4	-0.667	0.02765	0.0476	75
15	Thapsigargin	-0.496	3	-0.757	0.02934	0.2194	66
16	Pronetalol	0.265	4	0.657	0.03191	0.0089	50
17	Chloropyrazine	-0.328	4	-0.639	0.04048	0.0649	50
18	Naltrexone	-0.418	5	-0.576	0.04133	0.0899	60
19	Oxolamine	-0.355	4	-0.636	0.04255	0.1	50
20	Oxybenzone	-0.313	4	-0.635	0.04335	0.1268	50
21	Carisoprodol	-0.365	4	-0.633	0.04406	0.0248	50
22	Piperine	-0.393	4	-0.627	0.04782	0.0118	50

## Data Availability

The data used to support the findings of this study are included within the supplementary information files.

## Abbreviations

EMT: Epithelial-mesenchymal transition
RNA-seq: RNA-sequencing
TCGA: The Cancer Genome Atlas
GDC: Genomic Data Commons
TPM: Transcripts per kilobase million
GEO: Gene Expression Omnibus
GSEA: Gene Set Enrichment Analysis
GO: Gene Ontology
KEGG: Kyoto Encyclopedia of Genes and Genomes
RS: Risk score
LASSO: Least absolute shrinkage and selection operator
OS: Overall survival
ROC: Receiver operating characteristic
HR: Hazard ratio
CI: Confidence interval
CMap: Connectivity Map
ESTIMATE: Estimation of STromal and Immune cells in MAlignant Tumor tissues using Expression data.

## Supplementary Materials

Supplementary 1 Supplementary Table 1: a list of EMT-related gene signatures.

Supplementary 2 Supplementary Table 2: differentially expressed EMT-related genes in gastric cancer.

Supplementary 3 Supplementary Table 3: differentially expressed genes between high- and low-RS groups.
