# Supplementary material for: Discovery and Validation of an Epithelial-Mesenchymal Transition-Based Signature in Gastric Cancer by Genomics and Prognosis Analysis
Source: Biomed Res Int. 2021 Oct 26;2021:9026918. doi: 10.1155/2021/9026918 (PMC8570100; doi:10.1155/2021/9026918)
Supplement: Supplementary 2 — Supplementary Table 2: differentially expressed EMT-related genes in gastric cancer. [file 9026918.f2.pdf]

Supplementary table 2. Differentially expressed EMT-related genes in gastric cancer.

ID	logFC	AveExpr	t	P.Value
SFRP1	-2.652162623	2.318211174	-7.990776273	1.36E-14
MYL9	-1.940658479	7.593159793	-5.786282575	1.43E-08
ABI3BP	-1.683510092	3.106029756	-5.50130682	6.64E-08
TAGLN	-1.590284054	7.058879802	-4.658722586	4.30E-06
PCOLCE2	-1.441558057	1.306697229	-7.272295468	1.80E-12
MYLK	-1.370729835	5.121911294	-3.998059087	7.57E-05
FBLN1	-1.322985744	6.378227853	-3.934779915	9.77E-05
TPM2	-1.28067254	7.14913488	-4.165063841	3.79E-05
MFAP5	-1.149028531	3.472472557	-3.873129681	0.000124967
CXCL12	-1.13799746	4.289708005	-4.552485434	6.99E-06
DCN	-1.095691723	6.265357461	-4.314718514	2.00E-05
ACTA2	-1.086989152	8.202511084	-3.674677716	0.000269652
MSX1	1.052323717	2.697628438	5.809703015	1.25E-08
TGFBI	1.070032993	6.180035781	4.704298159	3.48E-06
FSTL3	1.110195996	4.451438229	4.565057456	6.60E-06
WNT5A	1.133099201	3.379308559	5.571369953	4.58E-08
LAMA3	1.185868368	4.224877242	4.517023425	8.20E-06
COL4A2	1.200738837	7.364642493	5.863827669	9.30E-09
LUM	1.217801995	7.833995158	4.854953498	1.71E-06
CALU	1.219795504	6.459115329	8.949858092	1.23E-17
LRRC15	1.24360677	2.070346181	4.901827342	1.37E-06
VEGFA	1.24410416	4.683477065	6.933965498	1.59E-11
PRRX1	1.249536696	3.594451479	4.895233537	1.41E-06
COL5A3	1.265716745	3.194959203	6.775123919	4.32E-11
NID2	1.295736081	3.727136252	6.265041489	9.41E-10
P3H1	1.394693628	4.563415013	10.88000908	2.12E-24
MMP14	1.410128369	7.05344789	7.781797792	5.84E-14
PVR	1.435807303	5.406890134	8.911523599	1.65E-17
DKK1	1.454605799	2.621650456	3.298793027	0.001055516
COLGALT1	1.468984062	5.855791206	12.28507277	9.12E-30
LOX	1.517777131	3.801500716	7.290973926	1.59E-12
MATN3	1.521051895	2.074658627	6.559111994	1.63E-10
CDH11	1.588355365	4.322004283	7.054776903	7.38E-12
BMP1	1.592866018	4.498428231	11.20873179	1.26E-25
COL7A1	1.621532739	2.737747649	6.707997398	6.54E-11
MCM7	1.656855667	6.251084663	9.969957003	4.10E-21
CXCL6	1.658417264	2.193241842	5.598857632	3.95E-08
ITGA2	1.661690638	4.395281836	8.213287943	2.82E-15
COL6A3	1.663161221	6.721663974	6.665794377	8.49E-11
FN1	1.671889764	7.763071168	5.241563171	2.55E-07
IGFBP3	1.718079399	7.236477412	7.895760114	2.65E-14
PDGFRB	1.725331353	5.735592941	8.114282912	5.70E-15
PLAUR	1.729771509	6.361942567	8.521863584	3.01E-16
IL32	1.731357779	7.464222364	7.701213161	1.02E-13
LOXL2	1.755429361	5.108185737	9.753014667	2.36E-20
TNFRSF12A	1.763406907	5.985473256	8.954590261	1.19E-17
TNFRSF11B	1.8018337	2.617577283	6.320386876	6.80E-10
PLOD3	1.824040402	6.457899655	11.63038193	3.16E-27
COL5A1	1.844667996	6.107576905	7.579537561	2.32E-13
SPARC	1.844930061	8.670151996	8.605107096	1.63E-16
SERPINH1	1.874926377	6.824129316	12.04382184	8.01E-29

VCAN	1.904105896	4.664317193	8.022767002	1.09E-14
MXRA5	1.914134996	5.381795753	7.906794421	2.45E-14
TIMP1	1.920030977	9.733531782	10.09686638	1.46E-21
COMP	1.928020819	2.578235496	5.370298455	1.32E-07
COL12A1	1.944376055	5.207415338	7.840642452	3.89E-14
ADAM12	1.986204817	2.213550067	8.835955221	2.92E-17
COL4A1	2.066698804	7.23463561	10.54054765	3.72E-23
THY1	2.067709718	5.763789825	9.571158534	1.00E-19
COL11A1	2.09821453	2.067897624	6.904573549	1.92E-11
PMEPA1	2.183523293	5.765772846	8.938580344	1.34E-17
SERPINE1	2.231793543	5.347155914	8.126500214	5.23E-15
COL5A2	2.239970974	5.620654951	10.09593044	1.47E-21
COL1A2	2.24114661	8.822003903	8.631379729	1.34E-16
FAP	2.258127381	3.068210083	9.345996763	5.87E-19
COL3A1	2.414574974	9.03327536	9.0697223	4.95E-18
MEST	2.466178497	5.172575314	12.13758179	3.45E-29
THBS2	2.521529225	5.100308915	8.002397044	1.26E-14
LAMC2	2.550186622	5.733617838	9.565690218	1.05E-19
MMP3	2.64555183	3.233279822	6.309857313	7.23E-10
SFRP4	2.784801196	4.590632111	6.63900623	1.00E-10
MMP1	2.84672022	5.605500409	7.107359365	5.26E-12
BGN	2.875285943	8.237813831	11.70875992	1.58E-27
CXCL8	2.971896465	5.479010059	7.981220915	1.46E-14
INHBA	3.023284501	3.898639917	12.55959716	7.52E-31
CXCL1	3.16376669	5.979897119	9.09669111	4.03E-18
COL1A1	3.282726757	9.525545655	11.85423758	4.35E-28
CTHRC1	3.364768706	5.355515357	12.82631595	6.51E-32
SPP1	3.413890902	6.105015229	8.827519775	3.11E-17

adj.P.Val	B
7.58E-14	22.48710794
4.39E-08	8.992732188
1.92E-07	7.509413144
1.00E-05	3.508077558
7.82E-12	17.717778
0.000152933	0.79016224
0.000189792	0.549858529
8.16E-05	1.44134247
0.000240321	0.319159895
1.57E-05	3.04561217
4.40E-05	2.04563368
0.000494774	-0.400403855
3.92E-08	9.117472176
8.20E-06	3.709414422
1.50E-05	3.099838049
1.35E-07	7.868148177
1.82E-05	2.89338509
2.95E-08	9.407377288
4.23E-06	4.387432567
1.12E-16	29.3528702
3.51E-06	4.602271807
6.37E-11	15.59147348
3.58E-06	4.571939132
1.66E-10	14.6207378
3.08E-09	11.62640769
4.23E-23	44.66079405
2.78E-13	21.06536187
1.37E-16	29.06818546
0.001773977	-1.66578684
6.08E-28	56.82365379
7.07E-12	17.83745155
5.71E-10	13.32952078
3.07E-11	16.34166032
2.80E-24	47.43837229
2.42E-10	14.21589334
5.86E-20	37.21680036
1.18E-07	8.009954411
1.88E-14	24.03098305
3.09E-10	13.96301769
6.89E-07	6.213818844
1.36E-13	21.83725206
3.57E-14	23.34026702
2.07E-15	26.22185687
4.72E-13	20.52456767
3.14E-19	35.49960268
1.12E-16	29.38806976
2.30E-09	11.94205456
7.91E-26	51.06419653
1.06E-12	19.71599306
1.16E-15	26.8225637
3.20E-27	54.68384696

6.60E-14	22.70718189
1.29E-13	21.91242812
2.26E-20	38.23197516
3.66E-07	6.849151312
1.94E-13	21.46289526
2.33E-16	28.50940435
6.76E-22	41.84042215
1.23E-18	34.07823998
7.52E-11	15.4105061
1.17E-16	29.26903265
3.37E-14	23.42517594
2.26E-20	38.22446011
9.94E-16	27.01299346
6.52E-18	32.34196682
4.95E-17	30.24828887
1.73E-27	55.5131145
7.40E-14	22.56697673
1.23E-18	34.03576105
2.41E-09	11.88182629
3.57E-10	13.80317141
2.24E-11	16.67134184
4.52E-26	51.74563335
7.89E-14	22.42149798
7.52E-29	59.28201584
4.24E-17	30.45084446
1.45E-26	53.0164182
1.30E-29	61.69321895
2.39E-16	28.44722933
