# Supplementary material for: Discovery and Validation of an Epithelial-Mesenchymal Transition-Based Signature in Gastric Cancer by Genomics and Prognosis Analysis
Source: Biomed Res Int. 2021 Oct 26;2021:9026918. doi: 10.1155/2021/9026918 (PMC8570100; doi:10.1155/2021/9026918)
Supplement: Supplementary 3 — Supplementary Table 3: differentially expressed genes between high- and low-RS groups. [file 9026918.f3.pdf]

Supplementary table 3. Differentially expressed genes between high-and low-RS groups.

ID	logFC	AveExpr	t	P.Value
SERPINE1	1.952945344	5.518095995	15.64822406	3.37E-42
MATN3	1.66816317	2.191825928	15.55899625	7.67E-42
LTBP2	1.379651281	4.700641549	12.11691882	1.89E-28
MFGE8	1.013651785	6.434078342	12.00446216	4.96E-28
VASN	1.065761817	4.141726997	11.8762766	1.48E-27
GLIS2	1.112984759	3.677506312	11.58556593	1.74E-26
LBH	1.046492684	5.305962947	11.51828006	3.07E-26
VCAN	1.36641312	4.783115002	11.47538198	4.40E-26
THBS1	1.464224917	6.412305072	11.44172366	5.84E-26
CCN2	1.246789117	7.302180429	11.34366829	1.33E-25
KIRREL1	1.042127868	3.758421694	11.31376031	1.70E-25
CERCAM	1.210771161	4.080055131	11.27771409	2.30E-25
TIMP2	1.182593998	7.199037899	11.16008541	6.11E-25
LRRC32	1.175962608	4.624085381	10.86320618	7.06E-24
SCARF2	1.254921717	3.872866524	10.85490163	7.55E-24
CMTM3	1.00912778	4.472513472	10.84966644	7.88E-24
CCN1	1.343678288	6.114272411	10.68024818	3.14E-23
EFEMP2	1.092013649	4.886607499	10.64320612	4.24E-23
ANTXR1	1.311441553	5.509314867	10.57037857	7.64E-23
MFAP2	1.377750765	3.982257054	10.56692943	7.85E-23
AEBP1	1.404772894	6.919584612	10.5496694	9.03E-23
MXRA8	1.381065188	5.969632574	10.52528101	1.10E-22
PDGFRB	1.133023678	5.868292145	10.47899355	1.60E-22
ITGA11	1.205425679	3.008706731	10.43585075	2.26E-22
BGN	1.316131688	8.472832125	10.35222448	4.41E-22
AXL	1.064314763	4.622861126	10.31901371	5.75E-22
GFPT2	1.17494685	2.70704427	10.30493339	6.43E-22
SPARC	1.124180139	8.789140904	10.25532293	9.55E-22
COL8A2	1.106353739	2.609423964	10.18625234	1.65E-21
GXYLT2	1.174063408	2.400290168	10.11822542	2.83E-21
CLDN11	1.134053066	1.934530103	10.11742435	2.85E-21
FSTL1	1.077730233	6.363177181	10.10776413	3.07E-21
COL8A1	1.541780772	4.232220101	10.06859473	4.19E-21
HEYL	1.140356136	3.906564474	10.06129801	4.43E-21
INHBA	1.258197721	4.129517301	10.01323628	6.47E-21
LOXL1	1.011464957	4.792208581	9.972760843	8.89E-21
MMP2	1.316854044	7.124544008	9.907791125	1.48E-20
FBN1	1.263656468	4.422429826	9.900915979	1.56E-20
GGT5	1.079220677	4.279175	9.858843697	2.17E-20
CDH11	1.144137059	4.423796399	9.855298477	2.23E-20
FN1	1.568761281	7.873318125	9.815592052	3.03E-20
LTBP3	1.025120419	5.716205827	9.809059152	3.19E-20
ADGRA2	1.066524398	4.333999452	9.774169676	4.18E-20
OLFML2B	1.276657737	4.514925902	9.758740292	4.71E-20
MYH10	1.087271065	3.69152503	9.740115076	5.44E-20
NTM	1.055384589	2.293348832	9.733847756	5.72E-20
FNDC1	1.707013241	3.752949876	9.722195721	6.25E-20
CLEC11A	1.03692815	3.991539144	9.687598769	8.17E-20
CRISPLD2	1.043617444	5.076586784	9.645499838	1.13E-19

BICC1	1.043034572	2.716726317	9.643392795	1.15E-19
EFEMP1	1.457379259	4.986494027	9.639792176	1.18E-19
CHST3	1.018248481	2.714959976	9.625651598	1.32E-19
MEDAG	1.191799464	2.722690091	9.59823745	1.63E-19
FAP	1.224836048	3.213650048	9.588666693	1.75E-19
THBS2	1.597600527	5.277038658	9.575382073	1.94E-19
MRC2	1.070323925	4.731954403	9.571995884	1.99E-19
TWIST1	1.054048827	2.270943708	9.548390012	2.38E-19
DEPP1	1.169975209	5.960247767	9.529722301	2.75E-19
SERPINF1	1.237881316	6.398117635	9.495025402	3.58E-19
COL5A2	1.091440243	5.778481407	9.472573864	4.25E-19
C1S	1.161023357	7.017838488	9.455861965	4.82E-19
PER1	1.012410178	4.491836833	9.444280511	5.27E-19
CTHRC1	1.322235086	5.603125499	9.443741544	5.29E-19
SLC2A3	1.029557974	4.088182925	9.400892977	7.33E-19
TIMP3	1.177063278	2.980585651	9.368989233	9.33E-19
ITGBL1	1.245581005	2.021177321	9.359278583	1.00E-18
FBLN2	1.393529688	4.407677303	9.334088128	1.21E-18
PTGIS	1.636892063	3.183092099	9.32299615	1.32E-18
C1R	1.085729598	6.594610471	9.300698684	1.56E-18
EVC	1.018693658	2.605979588	9.287557306	1.73E-18
COL5A1	1.171544292	6.254064853	9.279013247	1.84E-18
NNMT	1.142830502	5.487407063	9.254064181	2.22E-18
GEM	1.07265611	5.077248272	9.248712058	2.31E-18
CPXM1	1.160001847	3.591269144	9.216950883	2.93E-18
LOX	1.016532682	3.897018987	9.204034314	3.23E-18
SERPING1	1.144385624	7.752067411	9.157711821	4.57E-18
EHD2	1.016415367	6.178315717	9.146821244	4.96E-18
IGFBP3	1.045427424	7.347734881	9.13188852	5.54E-18
SULF1	1.428587613	5.874630554	9.089868771	7.58E-18
FGFR1	1.113527238	4.020435406	9.030509054	1.18E-17
ADAMTS2	1.193988563	4.177460027	8.988606537	1.61E-17
PODN	1.405941897	4.582293592	8.987400096	1.62E-17
FSTL3	1.154736429	4.576549803	8.986613005	1.63E-17
COL3A1	1.264683045	9.206853803	8.982765249	1.68E-17
C11orf96	1.11384038	5.750786891	8.973768375	1.79E-17
ELN	1.397021148	4.833953191	8.959468835	1.99E-17
CAVIN1	1.048866817	6.396361072	8.937421164	2.34E-17
COL1A2	1.231245657	8.986731325	8.926347088	2.54E-17
FBXO17	1.095843841	2.44716811	8.907817571	2.91E-17
CILP2	1.035031184	1.38188627	8.880151876	3.57E-17
PCOLCE	1.003759058	5.887806547	8.873447455	3.75E-17
LAMA2	1.002235228	2.35910777	8.837168188	4.89E-17
IGFBP6	1.035978462	4.544528943	8.82969552	5.16E-17
FSCN1	1.068477486	5.90101018	8.793317922	6.73E-17
DACT1	1.012413455	3.127454291	8.776568559	7.60E-17
RAB34	1.070488406	5.226312395	8.734076451	1.04E-16
SPOCK1	1.420313391	3.245952124	8.729293002	1.07E-16
MOXD1	1.142540502	3.220525494	8.716049751	1.18E-16
PALM	1.170818424	3.434228017	8.71399663	1.20E-16
TMEM119	1.101016043	4.008553167	8.674410002	1.60E-16
CTSF	1.074738531	4.146078834	8.668431005	1.67E-16
ISLR	1.417379711	5.683641471	8.653020582	1.86E-16

FBLN5	1.083293277	4.993429141	8.635363061	2.11E-16
HTRA3	1.130468808	5.334290751	8.610553334	2.53E-16
COL6A2	1.023531557	8.205715124	8.580988146	3.13E-16
LUM	1.117576143	7.895870225	8.557041628	3.71E-16
COL6A3	1.10574805	6.846166773	8.538846434	4.23E-16
FBLN1	1.550581646	6.259287217	8.482974546	6.31E-16
PLXDC2	1.000547939	3.453166242	8.482749083	6.32E-16
SFRP4	1.941019111	4.790825657	8.438798928	8.64E-16
SLC22A17	1.009185094	2.925349319	8.366086401	1.45E-15
COL1A1	1.246760324	9.794186941	8.341904787	1.71E-15
COL10A1	1.6933778	3.700516531	8.332791832	1.83E-15
COL12A1	1.074579248	5.348234446	8.306531023	2.20E-15
FKBP10	1.193219817	5.78291969	8.303096087	2.25E-15
MFAP5	1.309477395	3.357596503	8.293515908	2.41E-15
COMP	1.639080851	2.734713575	8.28871949	2.49E-15
DUSP1	1.011059703	7.336030662	8.26318603	2.98E-15
GPX3	1.062678655	5.705176353	8.251325518	3.24E-15
AKAP12	1.200934129	3.420065463	8.249766764	3.28E-15
ANGPTL2	1.032894511	5.219647542	8.239731038	3.51E-15
EMILIN1	1.06181472	5.702155739	8.231606137	3.72E-15
ROR2	1.082410459	2.735626043	8.196328028	4.76E-15
GAS1	1.268121744	2.951292276	8.189894862	4.98E-15
UCHL1	1.276508498	2.981128976	8.157714436	6.23E-15
CPE	1.156079439	4.128956873	8.069693861	1.15E-14
DCN	1.093346742	6.155547029	8.049976255	1.31E-14
DPYSL3	1.288956427	5.398598033	8.037410191	1.43E-14
GREM1	1.479447547	4.784621837	7.970793776	2.26E-14
SERPINE2	1.000548155	3.525156281	7.920313588	3.19E-14
CYBRD1	1.084926777	5.104531537	7.901648418	3.63E-14
SLIT2	1.021982424	1.92401033	7.866783298	4.60E-14
BCAM	1.023862957	5.501900114	7.856561772	4.93E-14
LDOC1	1.01365692	3.680750165	7.75731744	9.63E-14
FMOD	1.014290188	5.722314503	7.682959207	1.58E-13
MN1	1.027121555	2.473423452	7.637969324	2.14E-13
FOSB	1.374615793	4.31685137	7.532657602	4.30E-13
PRRX1	1.038861242	3.685587429	7.470508444	6.46E-13
MGP	1.355270627	7.403526079	7.468960758	6.53E-13
OBSL1	1.021217079	3.208340768	7.452011371	7.29E-13
SFRP2	1.961905961	5.660198841	7.419684501	9.01E-13
CYP1B1	1.259437854	3.017956455	7.398934562	1.03E-12
PRELP	1.419431752	3.558711816	7.292067499	2.06E-12
MMP11	1.312043723	4.857653806	7.285075614	2.16E-12
CCDC80	1.259130842	3.898833975	7.279934078	2.23E-12
COL14A1	1.175495154	3.969119213	7.260941646	2.52E-12
POSTN	1.053869856	6.805252688	7.172996955	4.42E-12
BOC	1.058931657	2.56505817	7.147591385	5.19E-12
SPON1	1.273058833	4.291237744	7.067436184	8.62E-12
CST2	1.163898247	2.672753201	6.998013999	1.33E-11
PDLIM3	1.10803903	4.435114494	6.965418629	1.63E-11
TNS1	1.12818154	5.456201115	6.959135254	1.70E-11
MSRB3	1.025784547	3.721893751	6.923853213	2.12E-11
AOC3	1.09310951	4.09263272	6.843114354	3.49E-11
SSC5D	1.029047591	3.208594773	6.809778383	4.28E-11

F13A1	1.125358414	2.791045234	6.800784773	4.52E-11
DKK1	1.663842824	2.765366605	6.676499962	9.64E-11
FLNA	1.050837078	8.166525103	6.590434953	1.62E-10
PCDH7	1.019165727	3.623831161	6.584319434	1.68E-10
LRRN1	1.012527273	1.615026195	6.580352598	1.72E-10
CRYAB	1.070624962	4.592980592	6.520318687	2.46E-10
ACTA2	1.037833876	8.118292007	6.49089439	2.93E-10
MFAP4	1.183895475	6.385369243	6.486554943	3.00E-10
TAGLN	1.206193358	6.953168989	6.438268541	4.00E-10
THBS4	1.56781865	3.453146863	6.39162784	5.25E-10
RSPO3	1.017320061	2.860223229	6.390095739	5.30E-10
SMOC2	1.152306675	4.759885465	6.328847468	7.58E-10
MYL9	1.142869715	7.466252858	6.313474412	8.29E-10
COL11A1	1.089382405	2.215450195	6.285341409	9.75E-10
SERPINA5	1.017858356	2.218904156	6.260208292	1.13E-09
CHRDL1	1.091132402	1.981854866	6.256853289	1.15E-09
C3	1.150617111	7.431804069	6.23650742	1.29E-09
APOD	1.513691915	5.9231248	6.13802445	2.27E-09
ASPN	1.029651838	5.020334855	6.081130687	3.13E-09
VSTM2L	1.021988785	2.000586918	6.024579501	4.30E-09
CPXM2	1.014033806	3.361564053	5.958345895	6.22E-09
L1CAM	1.069995363	2.306854219	5.90076081	8.56E-09
RBPMS2	1.007684457	3.164061254	5.797244097	1.51E-08
HPN	1.082108396	1.823265884	5.756705483	1.88E-08
HSPB8	1.034890387	3.953075273	5.729223946	2.18E-08
PEG10	1.125893234	2.320609697	5.612194497	4.07E-08
HSPB7	1.210491357	3.085560294	5.576911694	4.91E-08
LINC02381	1.001800798	4.115474117	5.570083515	5.09E-08
MYLK	1.042477948	5.026923597	5.54300291	5.87E-08
CLDN6	1.165408122	1.170847562	5.535935173	6.09E-08
SFRP1	1.022826286	2.107786122	5.418330084	1.12E-07
CHRDL2	1.211149335	4.064723373	5.326344588	1.80E-07
H19	1.271854602	4.445490922	5.266482168	2.44E-07
GPC3	1.091054961	3.243023567	5.192296113	3.53E-07
CRABP2	1.037674833	4.086104569	5.074941174	6.31E-07
BARX1	1.022293355	4.253273091	5.06680471	6.57E-07
PLN	1.001722816	3.201799772	4.944710041	1.19E-06
CST1	1.296884973	5.95418184	4.942472415	1.20E-06
LMOD1	1.052078127	4.066236412	4.901183335	1.46E-06
EEF1A2	1.076182469	2.092542697	4.789198257	2.48E-06
HSPB6	1.276970699	5.230726301	4.734441486	3.20E-06
IGF2	1.082413175	4.715993293	4.691118995	3.91E-06
AGR3	-1.076017932	6.200874979	-4.673203286	4.24E-06
FLNC	1.056049564	3.976301158	4.590822618	6.17E-06
CNN1	1.190914553	5.813863091	4.48878079	9.74E-06
C7	1.082546202	3.080627379	4.472177179	1.05E-05
GPA33	-1.035104567	3.503869769	-4.464028891	1.09E-05
OGN	1.012138524	3.005299523	4.371814469	1.63E-05
SYNPO2	1.021062426	4.061951786	4.161321539	3.99E-05
MYH11	1.190138852	6.453841163	4.056921169	6.14E-05
PCSK1N	1.115806688	3.67290658	3.93609965	1.00E-04
PHGR1	-1.19371951	6.043650057	-3.748614153	0.000208025
DES	1.226301296	6.328971618	3.409686917	0.000726435

PGC	1.187609259	6.980637751	2.688111885	0.007529886

adj.P.Val	B
1.86E-37	84.62681768
2.12E-37	83.82305601
3.48E-24	53.71017331
6.84E-24	52.76847167
1.64E-23	51.69940764
1.60E-22	49.2927504
2.42E-22	48.73938169
3.04E-22	48.38732035
3.58E-22	48.11149586
7.32E-22	47.31001171
8.54E-22	47.06616857
1.06E-21	46.77266824
2.59E-21	45.81788745
2.16E-20	43.42913755
2.17E-20	43.36276194
2.17E-20	43.32093145
8.24E-20	41.97259549
1.06E-19	41.67919543
1.60E-19	41.10384312
1.60E-19	41.0766437
1.78E-19	40.94060139
2.09E-19	40.74856651
2.84E-19	40.38472207
3.66E-19	40.04633772
6.57E-19	39.39248622
8.13E-19	39.13358107
8.87E-19	39.0239451
1.17E-18	38.63828314
1.94E-18	38.10298506
3.08E-18	37.57766595
3.08E-18	37.57149113
3.26E-18	37.49704937
4.20E-18	37.19560341
4.37E-18	37.13951826
6.05E-18	36.77065146
8.18E-18	36.460757
1.34E-17	35.96476963
1.39E-17	35.91238866
1.81E-17	35.59228442
1.83E-17	35.56534548
2.38E-17	35.26399937
2.41E-17	35.21448409
3.08E-17	34.95035717
3.42E-17	34.83371964
3.85E-17	34.69306194
3.99E-17	34.64576527
4.21E-17	34.55787835
5.24E-17	34.29727862
7.17E-17	33.98088454

7.20E-17	33.96506977
7.32E-17	33.93804928
8.07E-17	33.83198864
9.75E-17	33.62662491
1.04E-16	33.55500818
1.12E-16	33.45566935
1.12E-16	33.43036104
1.31E-16	33.25407495
1.50E-16	33.11484497
1.90E-16	32.85648452
2.19E-16	32.68959823
2.44E-16	32.56552558
2.63E-16	32.47961767
2.63E-16	32.47562127
3.55E-16	32.15833113
4.44E-16	31.92263888
4.70E-16	31.85099447
5.41E-16	31.66534655
5.83E-16	31.58369541
6.74E-16	31.41973239
7.27E-16	31.32320772
7.69E-16	31.26049452
9.09E-16	31.07756662
9.24E-16	31.038363
1.13E-15	30.80599684
1.23E-15	30.71163626
1.63E-15	30.37388992
1.71E-15	30.29463444
1.90E-15	30.18605561
2.50E-15	29.8811014
3.76E-15	29.45177099
4.92E-15	29.14974514
4.94E-15	29.14106217
4.94E-15	29.13539773
5.05E-15	29.10771106
5.37E-15	29.04300233
5.84E-15	28.94023759
6.80E-15	28.78198953
7.30E-15	28.70259632
8.28E-15	28.56989
9.74E-15	28.37207169
1.02E-14	28.3241909
1.27E-14	28.06548951
1.32E-14	28.01228583
1.64E-14	27.75369067
1.81E-14	27.63485211
2.37E-14	27.3340101
2.43E-14	27.30020157
2.64E-14	27.2066622
2.67E-14	27.19216875
3.45E-14	26.91314362
3.58E-14	26.87107142
3.92E-14	26.76271909

4.40E-14	26.63871918
5.21E-14	26.46476799
6.32E-14	26.25789554
7.34E-14	26.09067489
8.28E-14	25.96381863
1.17E-13	25.57537932
1.17E-13	25.5738152
1.51E-13	25.26943516
2.42E-13	24.76814092
2.81E-13	24.6020627
2.97E-13	24.53955793
3.49E-13	24.35969143
3.54E-13	24.33619264
3.77E-13	24.27068768
3.84E-13	24.23791083
4.57E-13	24.06363791
4.91E-13	23.98280871
4.95E-13	23.97219159
5.26E-13	23.90386755
5.50E-13	23.84859332
6.91E-13	23.60901826
7.21E-13	23.56540487
8.72E-13	23.34758522
1.50E-12	22.75476566
1.70E-12	22.62256662
1.84E-12	22.53843102
2.76E-12	22.09390638
3.76E-12	21.75875144
4.24E-12	21.63519931
5.22E-12	21.40495409
5.52E-12	21.33758623
1.02E-11	20.68666562
1.57E-11	20.2027734
2.03E-11	19.9115966
3.80E-11	19.23477231
5.52E-11	18.83850563
5.57E-11	18.82866762
6.12E-11	18.7210234
7.40E-11	18.51620752
8.38E-11	18.38507996
1.55E-10	17.71397406
1.61E-10	17.6703147
1.66E-10	17.63822902
1.85E-10	17.51985095
3.11E-10	16.9746665
3.61E-10	16.81808631
5.64E-10	16.3267768
8.30E-10	15.90459811
9.95E-10	15.70745354
1.03E-09	15.66952975
1.27E-09	15.45706136
1.99E-09	14.97392675
2.40E-09	14.77570258

2.51E-09	14.72235057
5.03E-09	13.99059718
8.02E-09	13.48996635
8.28E-09	13.4545844
8.45E-09	13.4316475
1.16E-08	13.0858339
1.36E-08	12.91724324
1.39E-08	12.89243016
1.79E-08	12.61720388
2.30E-08	12.35289093
2.32E-08	12.34423416
3.18E-08	11.9995068
3.44E-08	11.9133938
3.97E-08	11.75623508
4.53E-08	11.61630585
4.61E-08	11.5976605
5.13E-08	11.48475925
8.51E-08	10.9424207
1.14E-07	10.63226765
1.52E-07	10.32629006
2.13E-07	9.970868261
2.83E-07	9.664452894
4.65E-07	9.119751843
5.66E-07	8.908596922
6.46E-07	8.766147473
1.13E-06	8.165852336
1.33E-06	7.986892141
1.37E-06	7.952367166
1.55E-06	7.815788678
1.61E-06	7.780234744
2.76E-06	7.194215012
4.17E-06	6.743252904
5.43E-06	6.453291693
7.53E-06	6.097823104
1.26E-05	5.544328266
1.31E-05	5.506356351
2.22E-05	4.942876631
2.24E-05	4.932660781
2.66E-05	4.744876656
4.24E-05	4.242477051
5.31E-05	4.000519366
6.29E-05	3.810818076
6.77E-05	3.732816792
9.34E-05	3.377537839
0.000139546	2.945228354
0.000148997	2.87570235
0.000153749	2.841666076
0.000218389	2.460337508
0.000470897	1.616688877
0.000679547	1.212215949
0.001024481	0.755798634
0.001892533	0.0725857
0.005399504	-1.084122303

0.035037875	-3.20217415
